# Postnatal care provided by UK midwifery units and the impact of the COVID-19 pandemic: A survey using the UK Midwifery Study System (UKMidSS)

**DOI:** 10.1016/j.heliyon.2024.e29878

**Published:** 2024-04-24

**Authors:** Imogen Whyte, Alessandra Morelli, Ethel Burns, Rachel Rowe

**Affiliations:** a Oxford School of Nursing and Midwifery, Faculty of Health and Life Sciences, Oxford Brookes University, Oxford, United Kingdom; b NIHR Policy Research Unit in Maternal and Neonatal Health and Care, National Perinatal Epidemiology Unit, Nuffield Department of Population Health, University of Oxford, Oxford, United Kingdom

## Abstract

**Background:**

Postnatal care supports healthy transitions to parenthood, mother-infant relationships, and breastfeeding establishment. Highly valued by women and families, it is often an area where parents report low satisfaction compared with other areas of maternity care. Most research about postnatal care is hospital-focused. Little is known about postnatal services provided by midwifery units, and any changes to this provision since the COVID-19 pandemic.

**Aim:**

To describe postnatal care services provided by UK midwifery units and examine the extent to which provision was affected by the COVID-19 pandemic.

**Methods:**

We carried out a national survey online between January–June 2022 using the United Kingdom Midwifery Study System (UKMidSS). We asked about postnatal care provision in alongside midwifery units (AMU) and freestanding midwifery units (FMU), before the COVID-19 pandemic (July–December 2019) and shortly after restrictions were eased (January–June 2022).

**Findings:**

Overall 131 (67 %) midwifery units responded to the survey, 76 (62 %) AMUs and 55 (75 %) FMUs, from 75 % of eligible NHS organisations. In 2022, 66 % of AMUs reported that women typically stayed for 6–24 h after a straightforward birth, while 70 % of FMUs reported typical postnatal stays of <6 h. For 2019, significantly more FMUs reported providing outpatient postnatal services compared with AMUs (98 % vs 57 %, p < 0.001). From 2019 to 2022 there were significant reductions in partners staying overnight in midwifery units (65 %–42 %, p < 0.001), and in the provision of outpatient postnatal breastfeeding groups (23 %–15 %, p < 0.01) and other postnatal groups (7 %–2 %, p = 0.02).

**Conclusions:**

The findings document the ways in which postnatal care provision differs between AMUs and FMU, with potential consequences for choice and experience for women. They are also congruent with evidence that maternity care was adversely affected by the COVID-19 pandemic, including a reduction in postnatal visiting for partners and in postnatal group support services.

## 1 Introduction

Postnatal care is essential for the wellbeing of mother and infant. In this time, women regain their health and adapt to motherhood, developing a bond with their babies which is fundamental for the development of secure attachment [1]. Despite this importance, postnatal care is often the sector of maternity services with the lowest levels of women's satisfaction [[2], [3], [4]]. In the UK, because of “scanty and inadequate” provision, it is often referred to as a ‘Cinderella service’ [5].

In the UK, maternity services are provided through a publicly funded universal healthcare system, the National Health Service (NHS), by NHS organisations called Trusts or Health Boards serving particular geographical areas. National clinical guidance for the NHS in the UK covers postnatal care for women and babies in the immediate postnatal period and the first eight weeks after birth [5]. Typical length of postnatal inpatient stay following a singleton vaginal birth in a health facility varies by country, with the UK average at 1.5 days in 2011 compared with, for example, Germany and France at three and four days respectively [6]. In England in 2019 around three quarters of women went home within 24 h of a spontaneous vaginal birth, classified by many countries as an ‘early postnatal discharge’ [7,8]. Evidence from a systematic review of 17 trials in high-income countries indicates that that this may be associated with an increased risk of postnatal neonatal readmission, with uncertainty about any impact on maternal readmission within 6 weeks of birth, and infant or maternal mortality [8].

The postnatal period offers a window of opportunity to positively impact the future health of mothers and babies [5,9]. In the UK, midwifery care is provided by the NHS following discharge home for a period of around ten days during which midwives continue to monitor wellbeing and offer advice, support and national screening services at home or in community clinics [5]. Beyond this, postnatal care is provided by health visitors. In addition to professional support, advice and screening, supportive partner involvement during the perinatal period can also have positive effects on women's early postnatal experiences and longer term wellbeing, including improved breastfeeding initiation and continuation rates [[9], [10], [11]].

Most UK-based research into postnatal care has focused on care in hospital obstetric settings [8,12,13], but in the UK around 15 % of women give birth in midwifery units (MUs), often also known as ‘birth centres’ [14]. In the UK and elsewhere, MUs are located either on the same site as a hospital obstetric unit (alongside midwifery unit, AMU) or located on a separate site (freestanding midwifery unit, FMU) [14,15]. Different types of MU in the UK vary widely in size (number of births), location and staffing models [14,16], and evolved in different ways. FMUs originally ‘evolved’ as an alternative to hospital obstetric unit (OU) birth in remote rural areas [[17], [18], [19]]. From the 1990s onwards some new FMUs opened in hospitals where OUs had closed [20,21], but since at least 2010, withdrawal of 24-h staffing of FMUs and loss of FMU postnatal services have been recorded, with concern that this may impact the overall quality of care provided [22]. More recently some purpose-built FMUs have opened, many as ‘pop-up’ units, typically open only when a woman is admitted in labour [23]. Overall the number of FMUs, and the number of women giving birth in FMUs, has remained largely unchanged since the early 2000s [14]. In contrast, the number of AMUs has increased rapidly since 2008, largely in response to explicit national guidance that NHS organisations should offer care in an MU as an option [24]. In 2019, the most recent year for which published data are available, there were 132 AMUs and 91 FMUs across 151 NHS organisations providing maternity care in England, Scotland and Wales, with 19 % of NHS organisations having no MUs [25].

Some UK FMUs, in addition to providing care for women in labour, function as a hub out of which community midwives provide postnatal care regardless of where women gave birth, including postnatal appointments, screening services, additional breastfeeding support and group services [26], but the scope and extent of outpatient postnatal services provided by MUs is unknown.

Against this background of a lack of evidence about the postnatal care provided by MUs, there is also a need to understand the impact of the COVID-19 pandemic on this provision. Maternity services reduced postnatal services, furthering women's poor satisfaction of postnatal care [27,28]. Changes to postnatal care included increased restrictions to visitors of inpatient postnatal women, reduced routine in-person postnatal contact, the introduction of virtual/remote routine appointments, increased use of auxiliary staff, and additional postnatal clinics rather than home visits [29]. Women who gave birth during the pandemic reported poorer mental health, fewer postnatal services, less support and a greater need for breastfeeding support, compared with before the COVID-19 pandemic [28,30,31]. Women also reported feelings of isolation and loneliness in the early postnatal period in hospital without partners being able to visit [30,32].

A priority-setting exercise carried out with maternity service-user organisations for the NIHR Policy Research Unit in Maternal and Neonatal Health and Care (PRU-MNHC) at the University of Oxford identified postnatal care provided by UK MUs as a priority for research. The study reported here aimed to describe the postnatal care services provided by UK MUs and examine the extent to which this provision was affected by the COVID-19 pandemic.

## 2 Methods

### 2.1 Study design

A national, cross-sectional online survey was carried out using the UK Midwifery Study System (UKMidSS), a national infrastructure for carrying out observational studies and surveys of practice in UK MUs comprising a network of midwife ‘reporters’ in each MU, and a research and administrative team at the National Perinatal Epidemiology Unit at the University of Oxford [33].

### 2.2 Data collection

Data for this study were obtained from three sources.

1) Data about postnatal care services were taken from an online survey. All 195 MUs contributing data to UKMidSS studies at the time of the survey (around 90 % of all UK MUs at the time) were invited to take part. This included a small number of MUs that were temporarily closed and not providing intrapartum care at the time of the survey. UKMidSS reporters in each MU received a survey invitation by email. UKMidSS reporters are typically midwives who work in or have managerial responsibility for an MU; a small number are research midwives with a remit to support midwifery or maternity research. The invitation email, containing a unique access hyperlink to the survey hosted by the online platform LimeSurvey [34], was sent as a pilot to a small group of reporters on January 17, 2022 and to all remaining reporters on February 14, 2022. Up to six reminder emails, at fortnightly intervals, were sent to non-respondents.
2) Data about MU type (AMU/FMU), associated NHS Trust or Health Board (i.e. the NHS organisation in which each MU was based), and geographical location of the units were drawn from the UKMidSS administrative system.
3) Data about the number of births in each unit was drawn from the UKMidSS Postpartum Haemorrhage (PPH) Study, a national case control study investigating risk factors for PPH occurring in MUs, for which data collection took place in September 2019–February 2020 [35].

### 2.3 Survey instrument design

The postnatal care survey instrument (see supplementary file) was designed by IW in consultation with EB, RR and AM, and was reviewed by the UKMidSS Steering Group, which comprises senior midwives, obstetricians, a neonatologist, academics and two lay members.

Survey questions covered the following topics: typical length of postnatal stay following a straightforward birth; reasons for extended postnatal stay following a straightforward birth; circumstances of MU postnatal stay following birth in an obstetric unit; visiting hours and facilities for partners; and outpatient postnatal services offered by the MU. The survey included 12 closed questions, with single or multiple-choice response options, and two open-ended questions. Reporters were asked to respond to each question for two different time periods: before the COVID-19 pandemic, specifically within the last 6 months of 2019 (referred to below as “2019”); and at the time of the survey in January–June 2022 (referred to below as “2022”). Reporters in MUs that were temporarily not providing intrapartum care at the time of the survey were asked to complete questions about postnatal services before the COVID-19 pandemic and about any outpatient postnatal services at the time of the survey. Final free-text questions provided space for any further information about the impact of the COVID-19 pandemic on postnatal services.

### 2.4 Data analysis

Data were exported to SPSS version 28 [36] and STATA version 15.1 [37] for analysis. All completed responses were included in the dataset for analysis, irrespective of whether all survey questions were answered.

To assess for response bias, Pearson's Chi-square test was used to compare responding and non-responding units by type of MU (AMU/FMU), annual number of births, and nation of the UK (England, Scotland, Wales and Northern Ireland).

Descriptive statistics (frequencies and percentages) and Chi-square tests were used to describe and compare postnatal care services provided. Pearson's Chi-square was used to compare responses from different types of unit, with Fisher's exact test used when indicated (n < 5). McNemar-Bowker test of symmetry was used to compare MUs' responses between the two time points (2019 and 2022). A p-value of p < 0.05 was considered to be statistically significant.

## 3 Results

### 3.1 Survey response

**Fig. 1:**
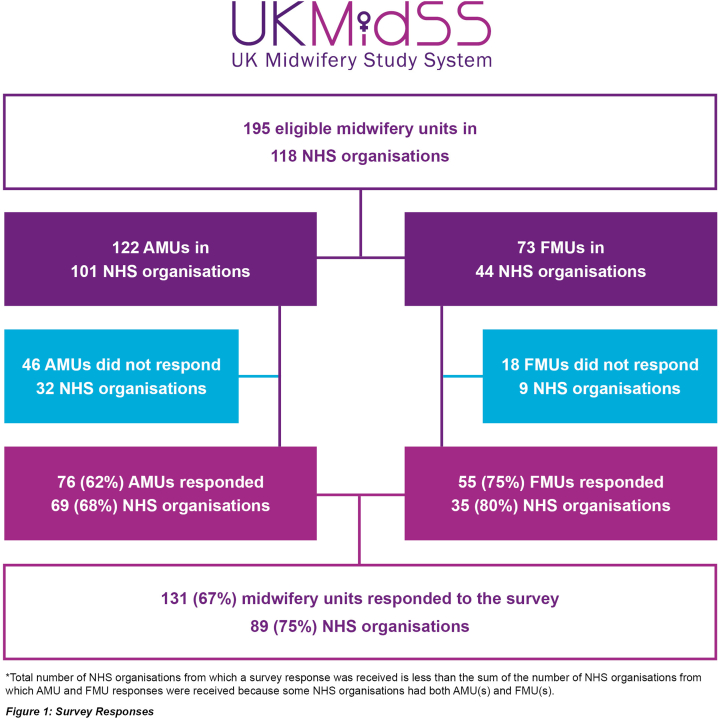
Survey response.

Overall, 195 MUs (122 AMUs and 73 FMUs) contributing data to UKMidSS, located in 118 NHS organisations (NHS Trusts and Health Boards), were eligible for inclusion in this study. Responses were received from 131 MUs (67 %) (Fig. 1), representing 75 % of all NHS organisations with MUs. Of the units who responded to the survey, 7 (5 %) were closed for intrapartum care in 2022, but responded to questions about care in 2019 and any outpatient postnatal care services. The response rate was similar in different types of MU (AMU vs FMU, p = 0.07) (Table 1). A smaller percentage of units from Northern Ireland responded (22 %), compared with England (65 %), Scotland (91 %) and Wales (76 %) (p < 0.001). A higher percentage of units with 100 births per year or fewer responded, compared with units with more births per year (p < 0.001).

**Table 1:** Characteristics of responding and non-responding units.

	Responded		No response		Total n = 195	P value^a^
	n = 131		n = 64			
	n	%	n	%		
**Type of unit**						0.067
AMU	76	62	46	38	122	
FMU	55	75	18	25	73	
**Nations of the UK and English regions**						<0.001 b
England	93	65	50	35	143	<0.001 c
London	18	64	10	36	28	
Midlands	21	55	17	45	38	
North	23	70	10	30	33	
South	31	70	13	30	44	
Northern Ireland	2	22	7	88	9	
Scotland	20	91	2	9	22	
Wales	16	76	5	24	21	
**Annual number of births**^d^						<0.001
≤100	32	78	9	22	41	
101-500	47	63	28	37	75	
501-1000	43	68	20	32	63	
≥1000	5	63	3	37	8	

a Pearson's Chi-square test.

b Comparing UK nations.

c Comparing English regions and other UK nations.

d Data drawn from 2019 to 2020 UKMidSS Postpartum Haemorrhage Study. Missing data for 8 units.

## 4 Survey findings

### 4.1 Length of postnatal stay and circumstance of extended stay

The survey asked respondents to state the typical length of postnatal stay after a straightforward vaginal birth. For 2019, just over half of responding MUs (56 %) reported that women would typically stay in the MU for 6–24 h after a straightforward vaginal birth, with just over one third (37 %) reporting shorter postnatal stay (Table 2). Typical length of postnatal stay in FMUs was shorter compared with AMUs (p < 0.001). Over half (55 %) of FMUs reported that women typically went home within 6 h of birth, compared with around a quarter of AMUs (24 %). Most AMUs (72 %) reported a typical postnatal stay of 6–24 h. Just over half (53 %) of MUs reported that, in some circumstances, women might be offered an extended postnatal stay, with no difference between AMUs and FMUs (p = 0.14) (Table 2). The most common reason for an extended postnatal stay in both types of unit in 2019 and 2022 was additional breastfeeding support, with a higher proportion of women receiving an extended postnatal stay for this reason in AMUs compared with FMUs in 2019 (p = 0.04).

**Table 2:** Typical length of postnatal stay in midwifery units.

	2019							2022							
	AMU		FMU		Overall		P value (AMU vs FMU)	AMU		FMU		Overall		P value (AMU vs FMU)	P value (overall 2022 vs 2019)
	n = 76		n = 55		N = 131			n = 74		n = 50		n = 124			
	**n**	**%**	**n**	**%**	**n**	**%**		**n**	**%**	**n**	**%**	**n**	**%**		
**Length of postnatal stay**							<0.001							<0.001	0.06
<6 h	18	24	30	55	48	37		22	30	35	70	57	46		
6–24 h	55	72	18	33	73	56		49	66	12	24	61	49		
24–48 h	3	4	5	9	8	6		3	4	2	4	5	4		
Other	0		2	4	2	2		0	0	1	2	1	1		
Closed	0		0		0	0		2		5		7			
**Extended postnatal stay**							0.14							0.02	0.41
Yes	44	59	25	44	69	53		43	59	19	38	62	51		
No	31	41	30	55	61	47		30	41	31	62	61	49		
Closed	0		0		0			2		5		7			
Missing	1		0		1			1		0		1			
**Circumstances of extended stay**^a^															
Mental health concerns	11	25	9	36	20	29	0.33	13	30	7	37	20	32	0.61	1.00
Breastfeeding support	42	96	20	80	62	90	0.04	38	88	15	79	53	86	0.33	1.00
Safeguarding concerns	18	41	7	28	25	36	0.28	21	49	8	42	29	47	0.62	0.25
Other b	18	24	12	48	30	43		21	49	9	47	30	48		

a Percentages calculated as a proportion of those offered extended postnatal stay.

b Other included: concerns about mother or baby's wellbeing, neonatal observations, awaiting Newborn and Infant Physical Examination (NIPE) screening, staffing pressure and bed capacity on postnatal ward.

For 2022, there was some indication that shorter lengths of stay (less than 6 h) had become more common in AMUs and FMUs, but there was no statistically significant difference between overall lengths of postnatal stay in 2022 compared with 2019 (p = 0.06) (Table 2). Again, more FMUs reported a typical length of stay of less than 6 h, compared with AMUs (70 % vs 30 %). A smaller proportion of FMUs reported offering extended postnatal stays in 2022 compared with AMUs (38 % vs 59 %, p = 0.02).

**Fig. 2:**
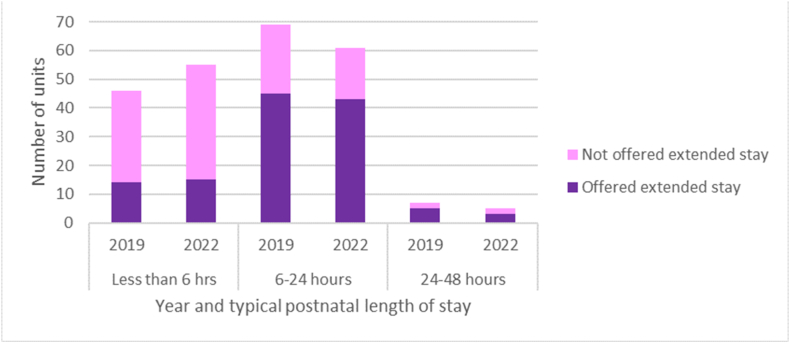
Offer of extended postnatal length of stay in midwifery units by typical length of postnatal stay, in 2019 and 2022.

In both 2019 and 2022, extended postnatal stays were more likely to be reported by units where the typical length of postnatal stay was more than 6 h (Fig. 2).

### 4.2 Postnatal stay in the midwifery unit following birth in an obstetric unit

We asked whether, and in what circumstances, a postnatal stay in the MU might be offered to women following birth in an obstetric unit (Table 3). FMUs were significantly less likely to offer a postnatal stay to women who had given birth on the obstetric unit compared with AMUs for both 2019 (29 % vs 57 %, p < 0.01) and 2022 (18 % vs 59 %, p < 0.001), with no significant difference between the two time periods (p = 0.79). The most frequently reported circumstances for postnatal care in the MU following birth in an obstetric unit for 2019 and 2022, were where the woman had originally planned to give birth in the MU (79 % and 75 % respectively), and when the postnatal ward was at full capacity (63 % and 75 % respectively) (Table 3). Postnatal ward capacity was more frequently reported as a reason for postnatal stays in AMUs compared with FMUs for 2019 (p < 0.001) and 2022 (p < 0.001). AMUs were less likely to report offering postnatal care for additional breastfeeding support following birth in an obstetric unit, compared with FMUs, in both 2019 (22 % vs 81 %, p < 0.001) and 2022 (24 % vs 78 %, p < 0.01).

**Table 3:** Postnatal care in midwifery units following birth in an obstetric unit.

	2019							2022							
	AMU		FMU		Overall		P value (AMU vs FMU)	AMU		FMU		Overall		P value (AMU vs FMU)	P value (overall 2022 vs 2019)
	n = 72		n = 55		N = 127			n = 71		n = 49		n = 120			
	**n**	**%**	**n**	**%**	**n**	**%**		**n**	**%**	**n**	**%**	**n**	**%**		
**Postnatal care following birth in an OU**							<0.01							<0.001	0.79
Yes	41	57	16	29	57	45		42	59	9	18	51	42		
No	31	43	39	71	70	55		29	41	40	82	69	58		
Closed	0		0		0			1		6		7			
Missing	4		0		4			4		0		4			
**Circumstances of postnatal care following birth in OU**^a^															
Planned midwifery unit birth	34	83	11	69	45	79	0.24	34	81	4	44	38	75	0.04	0.50
Postnatal ward bed capacity	33	81	3	19	36	63	<0.001	37	88	1	11	38	75	<0.001	0.50
Bereavement care	2	5	2	13	4	7	0.31	2	5	1	11	3	6	0.45	1.00
Mental health concerns	2	5	4	25	6	11	0.05	3	7	2	22	5	10	0.21	1.00
Breastfeeding support	9	22	13	81	22	39	<0.001	10	24	7	78	17	33	0.002	1.00
Safeguarding concerns	4	10	4	25	8	14	0.14	4	10	2	22	6	12	0.28	1.00
Other^b^	9	22	3	19	12	21		14	33	2	22	16	31		

a Percentages calculated as a proportion of those offered extended postnatal stay following birth in an obstetric unit.

b Other reasons included: neonatal observations, additional postnatal support (disability/surrogacy/maternal request/no partner), women having early discharge home, short staffing/postnatal ward capacity, and caring for COVID-19 positive women (2022 only).

### 4.3 Partner visiting and facilities

For 2019, 55 % of MUs responded that partners could visit at any time of day and 65 % responded that partners could stay overnight (Table 4). A higher proportion of AMUs reported that partners could stay overnight, compared with FMUs (78 % vs 48 %, p = 0.001). For 2022, compared with 2019, fewer MUs reported that partners could visit at any time (39 % vs 55 %, p < 0.001), or stay overnight (42 % vs 65 %, p < 0.001). More units reported that partners could visit only for a short period of time during the day in 2022, compared with in 2019 (11 % vs 1 %, p < 0.001). FMUs were more likely than AMUs to report that partners could visit for a set period of time after the birth, both for 2019 (35 % vs 9 %, p < 0.001) and 2022 (41 % vs 20 %, p = 0.02).

**Table 4:** Partner visiting and facilities.

	2019								2022							
	AMU			FMU		Overall		P value (AMU vs FMU)	AMU		FMU		Overall		P value (AMU vs FMU)	P value (overall 2022 vs 2019)
	n = 70			n = 55		N = 125			n = 69		n = 49		n = 118			
	**n**	**%**		**n**	**%**	**n**	**%**		**n**	**%**	**n**	**%**	**n**	**%**		
**Partner visiting hours**																
Set time after birth only	6	9		19	35	25	20^a^	<0.001	14	20	20	41	34	29^b^	0.02	0.04
Short visit (e.g. 11am-1pm)	1	1		0	0	1	1	1.00	9	13	4	8	13	11	0.55	<0.001
During daytime (e.g. 8am-8pm)	12	17		7	12	19	15	0.50	16	23	5	10	21	18	0.07	0.37
Anytime	41	59		28	51	69	55	0.39	26	38	20	41	46	39	0.73	<0.001
Overnight	53	78		24	48	77	65	<0.01	33	49	16	33	49	42	0.09	<0.001
Overnight in special circumstances	6	9		1	2	7	6	0.13	12	17	1	2	13	11	0.01	0.11
**Facilities available for partners**																
Double bed	26	37		6	11	32	26	<0.001	21	30	3	3	24	20	0.001	0.07
Pull-out bed	14	20		11	20	25	20	1.00	14	20	6	12	20	17	0.25	1.00
Recliner chair	28	40		16	29	44	35	0.21	23	33	16	33	39	33	0.94	0.34
Any sleeping facility	52	74		26	47	78	62	<0.01	42	60	19	39	61	52	0.02	<0.01
Meals offered	10	14		12	22	22	18	0.27	7	10	10	20	17	14	0.12	0.39
Drinks-making facilities	48	69		33	60	81	65	0.32	31	45	26	55	57	48	0.38	<0.001
Kitchen	21	30		22	40	43	34	0.24	7	10	14	29	21	18	0.01	<0.001

a Includes 13 units where the woman went home within 6 h of birth and partner could stay with her until discharge.

b Includes 11 units where the woman went home within 6 h of birth and partner could stay with her until discharge.

Fewer MUs reported providing sleeping facilities for partners in 2019 compared with 2022 (52 % vs 62 %, p < 0.01) (Table 4). AMUs were significantly more likely to offer any type of sleeping facility compared with FMUs for both 2019 (74 % vs 47 %, p < 0.01) and 2022 (60 % vs 39 %, p = 0.02). More AMUs (37 %) reported having double beds for couples to share, in comparison with FMUs (11 %) in 2019 (p < 0.001), and this difference remained for 2022. Fewer MUs reported providing partners with facilities to make drinks (65 % vs 48 %, p < 0.001), or access to a kitchen (34 % vs 18 %, p < 0.001), in 2022 compared with in 2019.

### 4.4 Outpatient postnatal services

FMUs were significantly more likely than AMUs to report offering the outpatient postnatal services listed in the survey, except for bereavement support where there was no statistically significant difference (p = 1.00) (Table 5). The differences between AMU and FMU services remained in 2022, with the exception of postnatal breastfeeding support groups (p = 0.27) and other lifestyle or social postnatal groups (p = 1.00). For both these types of group, the percentage of FMUs offering these in 2022 was lower than in 2019 (19 % vs 35 % and 2 % vs 13 % respectively). More AMUs reported offering no outpatient postnatal services compared with FMUs in 2019 (43 % vs 2 %, p < 0.001) and in 2022 (49 % vs 4 %, p < 0.001).

**Table 5:** Outpatient postnatal services offered by midwifery units.

	2019							2022							
	AMU		FMU		Overall		P value (AMU vs FMU)	AMU		FMU		Overall		P value (AMU vs FMU)	P value (overall 2022 vs 2019)
	n = 70		n = 55		N = 125			n = 70		n = 54		n = 124			
	**n**	**%**	**n**	**%**	**n**	**%**		**n**	**%**	**n**	**%**	**n**	**%**		
**Outpatient postnatal services**															
Routine postnatal appointments	14	20	49	89	63	50	<0.001	15	21	46	85	61	49	<0.001	1.00
NIPE	32	46	51	93	83	66	<0.001	25	36	48	89	73	59	<0.001	0.01
Hearing screening	16	23	28	51	44	35	<0.01	17	24	28	52	45	36	<0.01	0.69
SBR testing	8	11	25	46	33	26	<0.001	8	11	23	43	31	25	<0.001	0.50
Individual breastfeeding support	18	26	46	84	64	51	<0.001	18	26	41	76	59	48	<0.001	0.34
Breast pump rental	5	7	25	46	30	24	<0.001	4	6	22	41	26	21	<0.001	0.22
Breastfeeding support groups	10	14	19	35	29	23	<0.01	8	11	10	19	18	15	0.27	<0.01
Teenage mother support	4	6	10	18	14	11	0.04	2	3	7	13	9	7	0.04	0.13
Postnatal groups (lifestyle/social)	2	3	7	13	9	7	0.04	1	1	1	2	2	2	1.00	0.02
Bereavement care/support services	5	7	5	9	10	8	0.69	5	7	4	7	9	7	1.00	1.00
None	30	43	1	2	31	25	<0.001	34	49	2	4	36	29	<0.001	0.13
Other^a^	2	3	0	0	2	2		2	3	1	2	3	2		

NIPE = Newborn and Infant Physical Examination.

SBR= Serum bilirubin level.

a Anti-D outpatient administration, Tongue-tie division services, postnatal contraception services, online postnatal exercise class (2022).

## 5 Discussion

Our results showed significant differences between postnatal care services and facilities offered by different types of MU, and in 2022 compared with the last six months of 2019, before the COVID-19 pandemic.

In 2019, more than two thirds of AMUs reported a typical postnatal stay of 6–24 h, while postnatal stays in FMUs were typically shorter. Over half of FMUs reported that women typically went home within 6 h of birth, compared with only a quarter of AMUs. Relatively few MUs offered a stay of longer than 24 h, but there was some indication that this was more likely in FMUs compared with AMUs. MUs where women went home shortly after birth were less likely to offer an extended postnatal stay for women who might need it including, for example, for breastfeeding support. Reflecting their increased flexibility around postnatal stays beyond the immediate 6-h period after birth, AMUs were more likely than FMUs to offer postnatal care following birth in an OU, and also provided for longer visiting hours for partners, with evidence that they also had more varied facilities for partners in the postnatal period. The differences between AMUs and FMUs in relation to immediate postnatal care provision shown by the survey may in part reflect the different history, form and perception of AMUs and FMUs as places of birth [38], but may also be reflective of their changing relative positions within the landscape of UK maternity care provision [21].

The philosophy of care in FMUs typically supports flexible, woman-centred care, including extended postnatal support, community midwifery and outpatient postnatal services [21,39]. Our results, showing that FMUs are more likely than AMUs to support both shorter and longer typical postnatal stays, may be indicative of that flexibility. However, the finding that in over half of FMUs women typically only stay for up to 6 h after birth may also be considered to fit with a view that FMUs are increasingly ‘marginalised’ in maternity services [21], providing for example, more limited ‘intrapartum only’ staffing and care. This model of care offers those women who want it the option to return home quickly, but it also has a potential impact on the flexibility to provide extended postnatal support, should that be required, for example for women who have mental health concerns or who need more support with breastfeeding. Finally, if women only stay for the immediate post-birth period, then fewer facilities are required for birthing partners and visitors. This was also reflected in our findings, with FMUs less likely than AMUs to offer an overnight stay for partners and less likely to provide a place for partners to sleep. Further research into the length of postnatal stay in midwifery units and maternal and neonatal outcomes, including experience, may be of value.

Access and facilities for partners was one area where there was notable change between before and after the COVID-19 pandemic, across AMUs and FMUs, with just over a third of partners allowed to visit at any time in 2022, compared with over half before the pandemic. When partners were able to visit in 2022, they were less likely to have access to any sleeping facility, drinks making facilities and kitchen. This is likely to have reflected national guidance which restricted access to hospital facilities and visitors at the time [40].

Our findings around outpatient postnatal services illustrate the range of services provided by FMUs in this regard, and hence their potential value as a community service, particularly in rural areas, but also show the impact of the pandemic on those services. Nearly all FMUs that responded to our survey provided some outpatient postnatal services in 2019, while 40 % of AMUs did not provide any outpatient postnatal care and those that did offered a narrower range of services. This reflects the fact that in AMUs most postnatal services are provided before discharge or will be provided by community midwives, while most FMUs also provide community midwifery, including outpatient postnatal service provision. This is in line with other research and national recommendations about midwifery care being centred within community hubs [41]. While some of these services were maintained between 2019 and 2022, there was some evidence from our survey that group support services, for example for breastfeeding or general postnatal support, were adversely affected by the pandemic. The findings of decreased partner visiting hours and fewer postnatal groups are important in light of recommendations to support women and their families through the transition to motherhood [5,41], and given the vulnerability and importance of maternal mental health in the postnatal period [42].

Surveys conducted during the COVID-19 pandemic found that changes to the organisation of maternity care, although reduced compared to the initial first wave of COVID-19, continued to affect maternity services in the second wave of the pandemic [27]. In light of other research revealing changes to international midwifery and maternity care during the pandemic [[29], [30], [31]], our findings suggest that UK midwifery unit postnatal service provision had not yet recovered to a ‘pre-pandemic’ status in early 2022 when the survey was conducted. During the pandemic, the centralisation of maternity care included closure of midwifery units and redeployment of staff from midwifery units and community settings to hospitals against national guidance [27]. Our survey provides further evidence that in the UK, the reconfiguration of maternity services during the pandemic was not driven towards community-based care. Finally, ongoing systemic midwifery staffing shortages have the potential to further disproportionately adversely affect FMUs [21,43], despite potentially negative implications for women's choice and the availability of local services [41].

### 5.1 Strengths and limitations

This survey provides the first systematically collected evaluation of postnatal care provided by MUs, providing novel insight into postnatal care offered by MUs in the UK, before and after the COVID-19 pandemic. The use of the UKMidSS, a well-established research network, enabled a high response rate, which enhances validity and reliability of the findings, and there was minimal evidence of response bias.

The topic and research questions for this study were prioritised by maternity service-user representatives as part of a research priority-setting exercise. The results address questions of importance for women and their families, with implications for choice and a positive experience of birth and the postnatal period. They should also be of interest, therefore, to service providers and policymakers, particularly with a view to ensuring that services that are valued by women return to pre-pandemic levels. Involving service users in decision-making about their local MU, for example as recommended by international standards for midwifery units [44], would be one way of ensuring that the postnatal services provided reflect the needs of women and their families.

To compare services in 2022 with those during the pre-pandemic period we asked UKMidSS reporters to recall information about services that were provided in 2019. This is a potential source of recall bias, with the possibility that the proximity of the pandemic, and the disruption caused by it, may have resulted in them giving a more favourable impression of service provision before the pandemic. However, our data are largely in line with other data about postnatal care service provision during the COVID-19 pandemic.

Given the relatively small dataset, and with the data available to us, it was not possible to explore some further questions of interest, including for example whether the services provided varied according to rurality or number of births.

## 6 Conclusions

The findings from this survey describe the differences in postnatal care provision between different types of UK midwifery unit, with potential implications for women's choice and experience of care. We also document the impact of the COVID-19 pandemic on postnatal provision, including in particular a reduction in postnatal visiting for partners and in postnatal group support services. On the basis of the growing body of evidence about maternity service provision changes during the pandemic, relevant national and international stakeholders should work towards a return to pre-pandemic levels of care, and better preparedness and response for future outbreaks to minimise disruption to midwifery-led services.

## Funding

This research is funded by the 10.13039/501100000272National Institute for Health and Care Research (NIHR) Policy Research Programme, conducted through the Policy Research Unit in Maternal and Neonatal Health and Care, PR-PRU-1217-21202. The views expressed are those of the author(s) and not necessarily those of the NIHR or the Department of Health and Social Care.

## Ethics and consent declarations

Review and/or approval by an ethics committee was not needed for this study because the protocol was reviewed by the Joint Research Office study classification group at the University of Oxford, and it was deemed to be a survey of practice. As such, it was not subject to the Department of Health's UK Policy Framework for Health and Social Care Research (2017) [45] and research ethics review was not therefore required. Participants were informed that return of a completed survey questionnaire constituted consent to participate in the study.

## Data availability

Requests for access to the dataset underlying our findings will be considered by the National Perinatal Epidemiology Unit Data Sharing Committee and should be addressed to the data custodian, Professor Marian Knight (marian.knight@npeu.ox.ac.uk) in the first instance.

## CRediT authorship contribution statement

**Imogen Whyte:** Writing – review & editing, Writing – original draft, Project administration, Methodology, Investigation, Formal analysis, Data curation, Conceptualization. **Alessandra Morelli:** Writing – review & editing, Writing – original draft, Project administration, Formal analysis, Conceptualization. **Ethel Burns:** Writing – review & editing, Supervision, Conceptualization. **Rachel Rowe:** Writing – review & editing, Visualization, Supervision, Resources, Project administration, Methodology, Investigation, Funding acquisition, Conceptualization.

## Declaration of competing interest

The authors declare the following financial interests/personal relationships which may be considered as potential competing interests: Rachel Rowe reports financial support was provided by National Institute for Health and Care Research. Alessandra Morelli reports financial support was provided by National Institute for Health and Care Research. Rachel Rowe reports a relationship with National Institute for Health and Care Research that includes: funding grants. If there are other authors, they declare that they have no known competing financial interests or personal relationships that could have appeared to influence the work reported in this paper.
