# Supplementary material for: Postnatal care provided by UK midwifery units and the impact of the COVID-19 pandemic: A survey using the UK Midwifery Study System (UKMidSS)
Source: Heliyon. 2024 Apr 24;10(9):e29878. doi: 10.1016/j.heliyon.2024.e29878 (PMC11066328; doi:10.1016/j.heliyon.2024.e29878)
Supplement: Multimedia component 1 [file mmc1.docx]

**Supplementary file: Postnatal care survey instrument**

**Postnatal care provided by UK midwifery units:**

**a national survey of practice using the UK Midwifery Study System (UKMidSS)**

In this UKMidSS survey we are collecting information about postnatal care services provided by midwifery units (both alongside and freestanding) across the UK.

In response to the following questions please tell us about the postnatal care services provided in your midwifery unit before the COVID-19 pandemic, **specifically in the last 6 months of 2019**, and the postnatal care services that are provided **now**.

1. **Before the COVID-19 pandemic, in the last six months of 2019,** what was the typical length of postnatal stay **in your midwifery unit** for a woman who had a straightforward birth?

Choose one of the following answers.

- No postnatal stay in the midwifery unit – women typically go to a separate postnatal ward/area very soon after birth
- No postnatal stay in the midwifery unit – women typically go home very soon after birth
- <6 hours
- 6-24 hours
- 24 hours – 48 hours
- >48 hours
- Other (for example, if length of stay varies depending on time of day/night) Please specify

1. What is the typical **length of postnatal stay** in your midwifery unit **NOW** for a woman who has had a straightforward birth?

Choose one of the following answers.

- No postnatal stay in the midwifery unit – women typically go to a separate postnatal ward/area very soon after birth
- No postnatal stay in the midwifery unit – women typically go home very soon after birth
- <6 hours
- 6-24 hours
- 24 hours – 48 hours
- >48 hours
- Other (for example, if length of stay varies depending on time of day/night) Please specify

1. **Before the COVID -19 pandemic, in the last six months of 2019,** were there any circumstances in which a woman might have an **extended** postnatal stay **in your midwifery unit** after a **straightforward birth**?

Yes / No

If Yes, go to 3.1

3.1 What were the **circumstances** in which women might have an extended postnatal stay in your midwifery unit after a straightforward birth?

Check all that apply

- Mental health concerns
- Need for additional breastfeeding support
- Safeguarding concerns
- Other – please specify

1. Are there any circumstances in which a woman might have an **extended** postnatal stay **in your midwifery unit** after a **straightforward birth NOW**?

Yes / No

If Yes, go to 4.1

- 1. What are the **circumstances** in which women might have an extended postnatal stay in your midwifery unit after a straightforward birth **NOW**?

Check all that apply:

- Mental health concerns
- Need for additional breastfeeding support
- Safeguarding concerns
- Other – please specify

1. **Before the COVID -19 pandemic, in the last six months of 2019,** did your midwifery unit provide **inpatient** postnatal care for women who had an **instrumental birth in the midwifery unit**?

Choose one of the following answers

- Yes
- No
- Not applicable – no instrumental births in the midwifery unit

1. Does your midwifery unit provide **inpatient** postnatal care for women who had an **instrumental** birth **in** the midwifery unit **NOW?**

Choose one of the following answers

- Yes
- No
- Not applicable – no instrumental births in the midwifery unit

1. **Before the COVID -19 pandemic, in the last six months of 2019,** did your midwifery unit provide **inpatient** postnatal care for women **who gave birth in the obstetric unit/labour ward**?

Yes / No

If Yes, go to 7.1

7.1 What were the **circumstances** in which women who gave birth in the obstetric unit/labour ward might have received inpatient postnatal care in your midwifery unit **before the COVID -19 pandemic**?

Check all that apply:

- Women who planned to give birth in midwifery unit and were transferred to the obstetric unit during labour could return for postnatal care
- No postnatal beds in the obstetric unit/postnatal ward
- Bereavement care
- Mental health concerns
- Additional breastfeeding support
- Safeguarding concerns
- Other – please specify

1. Does your midwifery unit provide inpatient postnatal care for women who gave birth in the obstetric unit/labour ward **NOW**?

Yes / No

If Yes, go to 8.1

8.1 What are the **circumstances** in which a woman who gave birth in the obstetric unit/labour ward might receive inpatient postnatal care in your midwifery unit **NOW**?

Check all that apply.

- Women who planned to give birth in midwifery unit and were transferred to the obstetric unit during labour can return for postnatal care
- No postnatal beds in the obstetric unit/postnatal ward
- Bereavement care
- Mental health concerns
- Additional breastfeeding support
- Safeguarding concerns
- Other – please specify

1. **Before the COVID -19 pandemic, in the last six months of 2019,** what visiting/overnight stay facilities were provided for partners in the midwifery unit during the postnatal period?

Check all that apply.

- Partners could visit for a set amount of time after birth regardless of time of day (e.g. first 6 hours)
- Partners could visit at short set visiting hours only (e.g. 11am-2pm)
- Partners could visit anytime throughout daytime hours (e.g. 8am-8pm)
- Partners could visit at anytime, day or night
- Partners could stay overnight
- Partners could stay overnight in special circumstances only
- Double beds in some or all postnatal rooms for partners to share
- Pull out beds in some or all postnatal rooms for partners
- Recliner chairs in some or all postnatal rooms for partners
- Meals were provided for partners visiting/staying overnight
- Tea/coffee making facilities were available for partners
- Kitchen/food making facilities were available for partners
- Other – please specify

1. What visiting/overnight stay facilities are provided for partners in the midwifery unit during the postnatal period **NOW**?

Check all that apply.

- Partners can visit for a set amount of time after birth regardless of time of day (e.g. first 6 hours)
- Partners can visit at short set visiting hours only (e.g. 11am-2pm)
- Partners can visit anytime throughout daytime hours (e.g. 8am-8pm)
- Partners can visit at anytime, day or night
- Partners can stay overnight
- Partners can stay overnight in special circumstances only
- Double beds in some or all postnatal rooms for partners to share
- Pull out beds in some or all postnatal rooms for partners
- Recliner chairs in some or all postnatal rooms for partners
- Meals are provided for partners visiting/staying overnight
- Tea/coffee making facilities are available for partners
- Kitchen/food making facilities are available for partners
- Other – please specify

1. **Before the COVID -19 pandemic, in the last six months of 2019,** were any of the following **postnatal services** provided **in your midwifery unit** for women **after discharge home**?

Check all that apply.

- Routine postnatal appointments
- Newborn and Infant Physical Examination
- Newborn hearing screening
- Newborn serum bilirubin testing
- Individual breastfeeding support
- Breast pump rental
- Breastfeeding support group
- Teenage mother support
- Socialising / new parent / healthy lifestyle groups
- Postnatal bereavement care and services
- None of the above
- Other, please specify

1. Are any of the following **postnatal services** provided in your midwifery unit for women after discharge home **NOW**?

Check all that apply.

- Routine postnatal appointments
- Newborn and Infant Physical Examination
- Newborn hearing screening
- Newborn serum bilirubin testing
- Individual breastfeeding support
- Breast pump rental
- Breastfeeding support group
- Teenage mother support
- Socialising / new parent / healthy lifestyle groups
- Postnatal bereavement care and services
- None of the above
- Other, please specify

1. If postnatal services provided **in your midwifery unit** for women after discharge home are **different** **now** **compared to before the pandemic** in other ways, e.g. selected services that were face to face are now provided online, please explain that here.

Free text box

1. Is there anything else you would like to tell us about postnatal care/services provided **in your midwifery unit,** either before the pandemic or now?

Free text box

Thank you for completing this survey about postnatal care provided by UK midwifery units.

If you have experienced any problems completing this survey or wish to amend you results please email: ukmidss@npeu.ox.ac.uk
